# Complete Plastid Genome Sequence of the Basal Asterid *Ardisia polysticta* Miq. and Comparative Analyses of Asterid Plastid Genomes

**DOI:** 10.1371/journal.pone.0062548

**Published:** 2013-04-30

**Authors:** Chuan Ku, Jer-Ming Hu, Chih-Horng Kuo

**Affiliations:** 1 Institute of Plant and Microbial Biology, Academia Sinica, Taipei, Taiwan; 2 Institute of Ecology and Evolutionary Biology, National Taiwan University, Taipei, Taiwan; 3 Molecular and Biological Agricultural Sciences Program, Taiwan International Graduate Program, National Chung Hsing University and Academia Sinica, Taipei, Taiwan; 4 Biotechnology Center, National Chung Hsing University, Taichung, Taiwan; Wuhan Botanical Garden, Chinese Academy of Sciences, China

## Abstract

*Ardisia* is a basal asterid genus well known for its medicinal values and has the potential for development of novel phytopharmaceuticals. In this genus of nearly 500 species, many ornamental species are commonly grown worldwide and some have become invasive species that caused ecological problems. As there is no completed plastid genome (plastome) sequence in related taxa, we sequenced and characterized the plastome of *Ardisia polysticta* to find plastid markers of potential utility for phylogenetic analyses at low taxonomic levels. The complete *A*. *polysticta* plastome is 156,506 bp in length and has gene content and organization typical of most asterids and other angiosperms. We identified seven intergenic regions as potentially informative markers with resolution for interspecific relationships. Additionally, we characterized the diversity of asterid plastomes with respect to GC content, plastome organization, gene content, and repetitive sequences through comparative analyses. The results demonstrated that the genome organizations near the boundaries between inverted repeats (IRs) and single-copy regions (SCs) are polymorphic. The boundary organization found in *Ardisia* appears to be the most common type among asterids, while six other types are also found in various asterid lineages. In general, the repetitive sequences in genic regions tend to be more conserved, whereas those in noncoding regions are usually lineage-specific. Finally, we inferred the whole-plastome phylogeny with the available asterid sequences. With the improvement in taxon sampling of asterid orders and families, our result highlights the uncertainty of the position of Gentianales within euasterids I.

## Introduction

Plastids are crucial organelles for photosynthesis and other metabolic pathways, which arose only once through endosymbiosis of free-living cyanobacteria within eukaryotic cells [1]. The plastid genomes (i.e., plastomes) are valuable sources of phylogenetic information due to their relatively stable genome structure and higher evolutionary rate compared to mitochondrial genomes [2]. To date, over 170 complete angiosperm plastomes have been sequenced (NCBI Organelle Genome Resources). However, the taxon sampling of these sequences is highly uneven. For the two major eudicot clades, rosids have 75 complete plastomes available and asterids have only 36 (as of December 2012). In terms of the order-level lineages recognized in the Angiosperm Phylogeny Group (APG) classification system III [3], only five out of the 14 asterid groups have completed plastomes. In addition, several of the complete asterid plastomes were sampled from multiple species of the same genus (e.g., *Nicotiana*, *Solanum*) or even subspecies of a single species (*Olea europaea*). Furthermore, five of the 36 available asterid plastomes were sampled from parasitic lineages (*Epifagus virginiana* and *Cuscuta* spp.), which have undergone genome reduction and exhibit accelerated sequence divergence [4], [5].

To improve our understanding of plastome evolution and to expand taxon sampling in asterids, we chose the coral berry *Ardisia polysticta* Miq. for whole-plastome sequencing in this study. *Ardisia* is a member of the basal asterid order Ericales, which is the sister group to all euasterids [6]. It is one of the largest genera in the family Myrsinaceae [7] (or included in Primulaceae based on APG III [3]), estimated to have nearly 500 species distributed in the paleotropical and neotropical regions [8]. Fruits and other parts of the plant bodies are consumed for their nutritional values in Asia [9], [10], [11]. Additionally, many species are commonly used in traditional Chinese medicine to treat symptoms such as coughing and diarrhea [11]. Phytochemical studies have shown various medicinal properties of this genus, including antioxidant [12], anti-HIV [13], and anti-tumor [14] effects. Compounds with biological activities have also been identified from *Ardisia*, such as ardisicrenosides [15], ardisiaquinones [16], and ardisiphenols [17], indicating the potential for development of novel phytopharmaceuticals [18]. In addition to the nutritional and medicinal values, *Ardisia* also includes many well-known ornamental species cultivated worldwide (e.g., *A*. *japonica*, *A*. *crispa*, *A*. *squamulosa*, *A*. *escallonioides*). Among them, *A*. *crenata* has the longest history of cultivation – nearly 200 years since its first description as an ornamental [19]. It also has attracted great attention for being an invasive species in the USA [20]. The species chosen in this study, *A*. *polysticta*, is closely related to *A*. *crenata* according to molecular phylogenetic inference [21]. Both species are widely distributed, with *A*. *polysticta* mainly in Southeast Asia [22] and *A*. *crenata* East Asia [8]. Due to their morphological similarities, misidentification between these two species is a common problem [23].

In addition to offering a basal asterid reference for plastome comparisons within asterids, the complete *Ardisia* plastome will also be important for future studies on the plastid biology, plastid engineering, and phylogenetics of *Ardisia* and related genera. Plastids are the compartments for one of the two synthesis pathways of isopentenyl diphosphate in plants, which is converted into isoprenoids, steroids, terpenoids, and other compounds [24]. Many of the biologically active compounds isolated from *Ardisia* are saponins, which are glycoside derivatives of steroids or terpenoids [25] and are synthesized within plastids [26]. A fully sequenced plastome not only adds to our knowledge of *Ardisia* plastids, but also facilitates development of plastid genetic engineering in *Ardisia*, which could be used to increase the production of biologically active metabolites synthesized within plastids. Plastid transformation also has several advantages over nuclear transformation, including polycistronic gene expression, higher expression levels, and transgene containment due to lack of pollen transmission [27].

As a valuable resource for evolutionary analyses, the completely sequenced plastome could facilitate phylogenetic studies at lower taxonomic levels. Phylogenetic analysis using the *trnL*-*trnF* region, one of the most popular plastome markers for molecular phylogenetics, resulted in a largely polytomous tree of 12 *Ardisia* species [28]. To resolve interspecific relationships in the speciose genus *Ardisia*, the complete plastome can provide a reference for designing *Ardisia*-specific primers that amplify fast evolving regions reported for other angiosperms. Furthermore, it is known that different plastome regions show variable rates of evolution across plant taxa [29] and it is difficult to find a set of markers applicable to a wide range of plant lineages [30]. A solution to this problem would be to use the complete plastome sequence to identify *Ardisia*-specific fast evolving regions. In addition to resolving interspecific relationships, these markers could also be used for the identification of *Ardisia* species, which is difficult and causes confusion to researchers studying their medicinal usages [18]. Furthermore, the simple sequence repeats (SSRs) in plastomes can be used for evolutionary and ecological studies at the levels of cultivars, populations, and closely related species [31]. For example, the SSR markers can be used to track the population histories of closely related *A*. *polysticta* and *A*. *crenata*, which share much of their distribution range. These SSR markers can also supplement previous studies on the expansion history of invasive *A*. *crenata* populations such as that by Niu et al. [20], which used the largely invariable *trnL*-*trnF* as the only plastome marker. Due to their maternal inheritance, both the *Ardisia*-specific fast evolving regions and SSRs in the plastome could also assist in the characterization, parent identification, and selection of new cultivars of *Ardisia* ornamentals.

In this study, we determined the complete plastome sequence of *A*. *polysticta* and characterized its genome structure, gene content, and other characteristics such as repetitive sequences. Through comparative analysis with other asterid plastomes based on a phylogenetic framework, we aim to investigate the evolutionary history of plastomes in this major angiosperm clade. Furthermore, we examined the divergence level between *Ardisia* and euasterids in plastome intergenic regions to identify a list of molecular markers that can facilitate future phylogenetic studies.

## Materials and Methods

### Sequencing and Assembly

Fresh leaves were collected from *A*. *polysticta* at Yuanyang Valley, Hsinchu County, Taiwan. The voucher specimen (Ku028) was deposited in the National Taiwan University Herbarium (TAI). *A*. *polysticta* is not an endangered or protected species in Taiwan. According to the regulations of the Forestry Bureau (Council of Agriculture, Taiwan), no specific permits were required for collection of non-protected species at Yuanyang valley because this location is not a part of a nature reserve or national park.

For DNA extraction, 1.6 g of leaves was grounded using a ceramic mortar and pestle set with 15 mL PBS. The suspension was filtered through 100 µm filters and centrifuged at 1,200 g to remove uncrushed tissues and intact plant cells. The supernatant was then centrifuged at 16,000 g to pellet subcellular parts, from which DNA was extracted using the Tri-Plant Genomic DNA Reagent Kit (Geneaid, Taipei, Taiwan). A paired-end library was prepared from the DNA sample and sequenced using the HiSeq 2000 platform (Illumina, USA) by a commercial sequencing service provider (Yourgene, Taiwan). The Illumina sequencing technology was chosen because it is more accurate for sequencing homopolymers compared with Roche 454 platforms [32] and has been shown to work well for other plastomes [33], [34]. As many plastome SSRs are mononucleotide repeats with variable lengths in different haplotypes [31], it is important to accurately sequence these motifs. Approximately 224 million paired-end reads of 101 bp were obtained, with an average insert size of 251 bp. The raw reads were quality trimmed at the first position from the 5′-end that has a quality score of lower than 20. Reads that are shorter than 70 bp after the quality trimming were discarded.

For the *de novo* genome assembly, we used Velvet 1.2.07 [35]. The assembly parameters were set to k = 55, expected coverage = 1,500X, maximum coverage = 7,500X, and coverage cutoff = 300X based on our iterative optimization tests. To distingusih the scaffolds of plastid origin from those of nuclear or mitochodial, we used the BLAST [36], [37] similarity searches against the NCBI nr database [38] to identifiy scaffolds that encode plastid genes. Three large scaffolds that contain approximately 129 kb of unique sequence in the *A*. *polysticta* plastome were identified in the initial draft assembly. Primer walking and additional Sanger sequencing were then used to fill the gaps within and between these scaffolds and to validate the regions with possible assembly artifacts. The final complete plastome sequence was further checked by using BWA [39] for mapping all Illumina reads and IGV [40] for visual inspections.

### Annotation and Genome Map Drawing

The online automatic annotator DOGMA [41] was used to annotate the *A*. *polysticta* plastome. BLAST against other plastomes was also used to verify questionable regions in the DOGMA draft annotation. For tRNA genes, the annotations were also confirmed using tRNAscan-SE [42]. The annotations exported from DOGMA were compared with those of other plastomes and manually curated. The genome map and positions of repetitive sequences (see below) were drawn with the help of OGDRAW [43] and GenomeVx [44].

### Genome Analyses

To have a comprehensive overview of asterid plastome evolution, we compared the *A*. *polysticta* plastome with other available asterid plastomes (Table S1) with respect to GC content, genome organizations, and content of repetitive sequences. Because the intergenic regions are the most variable parts in plastomes [45], [46], we calculated the sequence divergence between *A*. *polysticta* and representative euasterids to find regions of potential phylogenetic utility for *Ardisia* at lower taxonomic levels. To avoid biases in mutation rate in the selected euasterid plastome, two plastomes with similar gene content and gene order to those of the *A*. *polysticta* plastome were chosen, including *Sesamum indicum* (euasterids I) and *Panax ginseng* (euasterids II). There are a total of 126 intergenic regions in the *A*. *polysticta* plastome (the 5′ and 3′ portions of *rps12* are considered different genic regions), of which 16 were IR duplicates. The 110 unique regions were parsed out from the three genomes using custom Perl scripts, aligned using MUSCLE [47] with the default settings, and analyzed using the DNADIST program in the PHYLIP package [48] to calculate the sequence divergence.

For characterization of repetitive sequences, the program Msatfinder v2.0 [49] was used to find SSRs in the plastomes of *A*. *polysticta* and other asterids by setting the minimum number of repeats to 10, 5, 4, 3, 3 and 3 for mono-, di-, tri, tetra-, penta- and hexanucleotides. For tandem and dispersed repeats, the program REPuter [50] was used to identify these elements with a repeat unit of at least 26 bp and sequence identity greater than 90%.

### Phylogenetic Analysis

We used plastome genes to reconstruct a phylogeny of asterids with completed plastomes (Table S1). Holoparasitic or hemiparasitic taxa that were previously reported to have accelerated evolutionary rates in plastomes [4], [5] were excluded from our analysis to avoid problems in phylogenetic reconstruction. In addition, *Parthenium argentatum* (Asteraceae) was also excluded due to the inconsistency in the number of protein-coding genes reported in the original study (85; Kumar et al. [51]) and the annotation found in the GenBank entry (56; accession number NC_013553). It is possible that the exclusive use of 454 reads in the assembly of this genome [51] has produced many frameshift artifacts. To avoid overrepresentation of certain genera or families, we reconstructed another tree in which only one plastome was included for each genus and at most two plastomes from different genera for each family. The protein-coding and rRNA genes were parsed from the selected plastomes of asterids and outgroups and clustered into ortholog groups using OrthoMCL [52]. We then examined the presence/absence of orthologous genes in each plastome. To confirm gene absence, we used the genic sequences of *A*. *polysticta* as the queries to run BLAST searches against the plastome in question. False negative results due to misannotation (e.g., *ycf1* in *Lactuca*, *rps19* in *Boea*, *infA* in *Olea*) were manually corrected to increase the number of usable genes for phylogenetic inference. In total, we included 74 protein-coding and four rRNA genes that are present in all of the plastomes analyzed. The sequences were aligned with MUSCLE with the default settings, concatenated into a single alignment of 77,976 characters, from which a maximum likelihood phylogeny was inferred using PhyML [53] with the GTR+I+G model and six substitution rate categories. Nodal supports were estimated from 1,000 bootstrap [54] samples of the alignment generated by the SEQBOOT program of PHYLIP.

## Results and Discussion

### Genome Organization and GC Content

**Figure 1:**
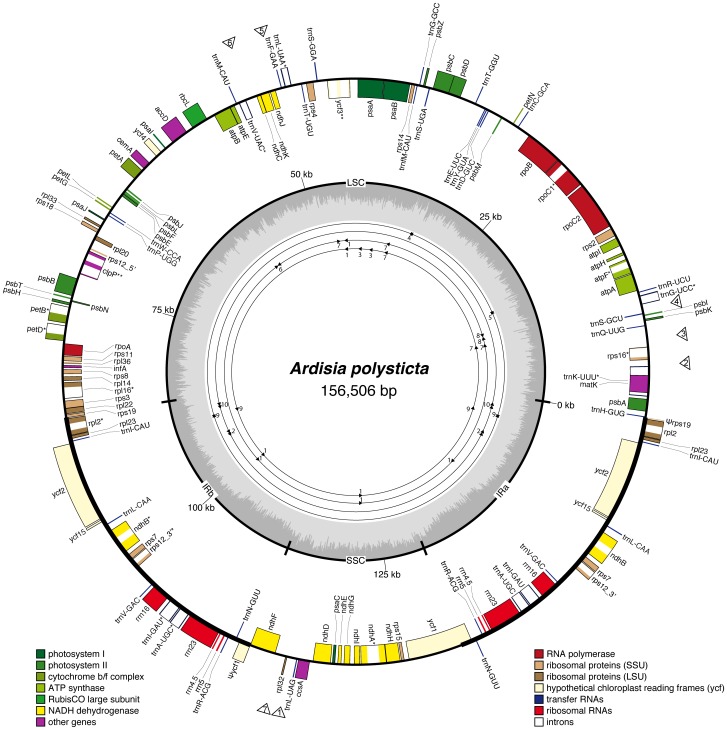
Plastome map of *Ardisia polysticta*. Genes drawn inside the circle are transcribed clockwise, those outside counterclockwise. The within-plastome GC content variation is indicated in the middle circle. Pseudogenes (Ψ) and genes containing one (*) and two (**) introns are indicated. Regions of potential phylogenetic utility (Table 2) are indicated by hollow triangles outside the circle (numbered from more to less divergent). Numbers and locations of repetitive sequences (Table 4) are drawn on the four inner circles (from inside: dispersed direct repeats (forward triangles), inverted repeats (forward and reversed triangles), tandem repeats (tandem triangles), palindromic sequences and a sequence that matches its reversed sequence (hexagrams)).

The complete plastome of *A*. *polysticta* (deposited in GenBank under the accession number KC465962) has a total length of 156,506 bp (Figure 1). It has a pair of inverted repeats (IRa and IRb) of 26,050 bp that separate a large single copy (LSC) region of 86,078 bp and a small single copy (SSC) region of 18,328 bp (Table 1). The genic regions account for 58.3% of the genome, including 86 protein-coding (50.7%), eight rRNA (5.8%), and 37 tRNA genes (1.8%) (Table S2). Six tRNA and ten protein-coding genes contain one intron and two protein-coding genes (*ycf3* and *clpP*) have two introns, while the remaining genes are intronless. The *rps12* gene, as in *Nicotiana* [55], consists of a 5′ portion (exon 1) in LSC and a 3′ portion (exons 2 and 3) in IR. The GC content of the whole plastome is 37.07%, with the IRs having a higher GC content (43.01%) than those of LSC (34.94%) and SSC (30.17%) due to the presence of GC-rich rRNA genes (Figure 1).

**Table 1:** Base composition of the *Ardisia polysticta* plastome.

	G/C (%)	A (%)	T (%)	C (%)	G (%)	Length (bp)
Total	37.07	31.17	31.75	18.87	18.19	156,506
By chromosomal region						
LSC	34.94	32.01	33.04	17.92	17.01	86,078
IRb	43.01	28.54	28.44	20.76	22.24	26,050
SSC	30.17	34.87	34.95	15.82	14.34	18,328
IRa	43.01	28.44	28.54	22.24	20.76	26,050
By codon position						
Position 1	45.33	30.77	23.90	18.65	26.68	26,535
Position 2	37.77	29.58	32.65	19.95	17.81	26,535
Position 3	29.40	32.17	38.44	13.58	15.82	26,535

It is notable that the plastome sequence of the basal asterid *A*. *polysticta* has the second lowest GC content among all reported asterid plastomes (Table S1). The asterid plastome with the lowest GC content found so far is that of *Epifagus virginiana*, a holoparasitic plant with the second smallest land plant plastome reported to date (70,028 bp) [56]. Because of the mutational bias of GC-to-AT substitutions [57], [58], it is not surprising that the *Epifagus* plastome, characterized by accelerated evolution and extensive reduction [5], [56], has a GC content below the norm of asterids. This effect is also evident in the hemiparasitic genus *Cuscuta*, where the two more reduced plastomes (*C*. *gronovii*, *C*. *obtusiflora*) have lower GC contents than those of the two other less reduced plastomes. When *Epifagus* is not considered, GC contents of asterid plastomes fall within the range from 37.07% to 38.33%, which is relatively narrow compared with either rosids (33.97–39.61%) or monocots (36.65–39.01%) [2]. Additionally, the GC contents show little within-genus variation (*Nicotiana*: 37.79–37.88%, *Solanum*: 37.86–37.88%, *Olea*: 37.79–37.81%), indicating different lineages have specific ranges of GC contents. When compared with the outgroup *Spinacia* (Caryophyllales, 36.82%), there is a trend toward increased plastome GC content from the outgroup to the basal asterid and then to euasterids.

### Divergence of Intergenic Regions

To investigate the variation of sequence divergence rates among intergenic regions, we compared the *A*. *polysticta* sequences to that of *Panax ginseng* and *Sesamum indicum*. Subsequently, we identified the 20 most conserved and the 20 most divergent regions in these two comparisons. Among the most conserved regions, the two pairwise comparisons shared 17 homologous regions (Table 2). Twelve of these regions are in IRs, which is consistent with the observation that IRs are more conserved than LSC and SSC [45], [46], [59]. The other five regions are relatively short (<60 bp) and are located within polycistronic transcription units [60], [61]. The high levels of sequence conservation in these regions may be explained by selective constraint that stemmed from their roles in splicing.

**Table 2:** List of the most conserved and the most divergent intergenic regions in *Ardisia*-*Sesamum* and *Ardisia*-*Panax* comparisons.

Most conserved^a^		Most divergenta,b			
Region	Pairwise distance	Region	Pairwise distance	Length (bp) in *Ardisia*	Shaw et al. (2007)^c^
*ndhA-ndhH*	0.000	***rpl32-trnL-UAG***	0.556	790	✓
*rpoC1-rpoB*	0.000	*ndhG-ndhI*	0.456	341	
*psbL-psbF*	0.000	*rpl14-rpl16*	0.455	120	
*rpl2-rpl23*	0.000	*ccsA-ndhD*	0.430	249	
*rrn23-rrn4.5*	0.010	***trnK-UUU-rps16***	0.419	531	✓
*trnI-GAU-trnA-UGC*	0.016	*trnH-GUG-psbA*	0.394	425	
*trnV-GAC-rrn16*	0.037	***rps16-trnQ-UUG***	0.391	1,771	✓
*rrn16-trnI-GAU*	0.039	*trnL-UAG*-*ccsA*	0.376	100	
*psaB-psaA*	0.041	***trnS-GCU*** **-** ***trnG-UCC***	0.375	737	
*rrn4.5-rrn5*	0.045	*trnS-GGA-rps4*	0.374	305	
*ycf2-ycf15*	0.046	***trnF-GAA-ndhJ***	0.364	503	
*trnN-GUU-ycf1*	0.047	***ndhC-trnV-UAC***	0.354	967	✓
*trnI-CAU-ycf2*	0.047	*psbI-trnS-GCU*	0.339	62	
*rpl23-trnI-CAU*	0.057	***ndhF-rpl32***	0.334	902	✓
*ndhB-rps7*	0.058	*rps15-ycf1*	0.327	393	
*rps7-rps12_3'*	0.059				
*psbT-psbN*	0.073				

a Among the 20 most conserved/divergent regions in *Ardisia*-*Sesamum* and *Ardisia*-*Panax* pairwise comparisons, the shared regions are listed in the order of increasing/decreasing divergence in *Ardisia*-*Sesamum* comparison.

b The most divergent regions in boldface are those of potential phylogenetic utility (>500 bp).

c Regions highlighted in Shaw et al. (2007) are checked.

Among the most divergent regions, 16 were shared between the two pairwise comparisons. Surprisingly, several plastome markers commonly used for molecular phylogenetics of asterids at low taxonomic levels, such as regions between *trnT*-*UGU* and *trnF*-*GAA* [20], [28], [62], [63] and between *atpB* and *rbcL* [63], [64], [65], are not included in this list. To resolve interspecific relationships in a speciose genus such as *Ardisia*, suitable markers should be variable and, at the same time, encompass a region of adequate length, so that there will be sufficient characters. Therefore, we suggest that the intergenic regions of over 500 bp are markers of potential phylogenetic utility for *Ardisia*, as highlighted in Table 2 and Figure 1. Other regions may also contain useful information for phylogenetic analyses, but their utility is limited by the short sequence lengths. For example, the *trnH-GUG*-*psbA* spacer region is frequently used for phylogenetic analyses, but has an average length of only 465 bp and thus is often too short to yield a well-resolved phylogeny [66]. All of the seven highlighted regions are located in SC regions, with two in the region between *trnF-GAA* and *trnV-UAC* in LSC, three between *trnK-UUU* and *trnG-UCC* in LSC and the other two between *ndhF* and *trnL-UAG* in SSC (Figure 1). Among them, several were found to be highly variable in other studies: three in comparison between *Helianthus* and *Lactuca* [30] and four in comparison among olive cultivars [67]. Five of them were also found in the list of nine intergenic regions recommended for angiosperm molecular phylogenetics at low taxonomic levels [68], further indicating the potential of these regions for species-level phylogenetics of *Ardisia*. It is notable that the longest of the 16 regions, *rps16*-*trnQ*-*UUG*, has a length of only 429 bp in *Arabidopsis* and only 407 bp in *Spinacia*, which has a sister relationship with asterids [3]. In asterids, its length is mostly in the range between 800 and 1,300 bp, and is over 1,700 bp in three lineages distributed in euasterids I (Oleaceae), euasterids II (*Panax*) and basal asterids (*Ardisia*). This length variation suggests that this region evolves relatively fast in asterids. In addition, its length in *A*. *polysticta* is shorter than 1,800 bp and thus could be sequenced with a single PCR run and Sanger sequencing with primers at both ends. In light of these, the *rps16*-*trnQ*-*UUG* spacer appears to be the best candidate marker for resolving interspecific relationships in *Ardisia*.

### Simple Sequence Repeats (SSRs)

There are 57 SSRs with a length of at least 10 bp in the *A*. *polysticta* plastome, including 45 mono-, four di-, seven tetra- and one pentanucleotide repeats (Table 3). No trinucleotides or hexanucleotides were found. Most (43/45) of mononucleotides consist of A or T and all of the dinucleotides are AT or TA repeats, which is consistent with the AT-richness of the plastome. We also screened the other asterid plastomes for SSRs with a length of at least 10 bp (Table S1). The number of SSRs in each asterid plastome ranges from 27 in *Boea* to 75 in *Crithmum*. It is quite surprising that the largest number of plastome SSRs found in asterids is much smaller than the number of SSRs in the rosid *Arabidopsis thaliana* (104) or in the monocot *Dioscorea elephantipes* (95; NC_009601; 152,609 bp, 37.15% GC).

**Table 3:** Distribution of simple sequence repeats in the *Ardisia polysticta* plastome.

Repeat unit	Length (bp)	Number of SSRs	Start position^a^
A	10	6	5,337; 33,249; 43,327; 64,939; 83,012; 116,070
	11	3	4,628; 14,067; 112,074 (Ψ*ycf1*)
	12	3	22,968; 82,976; 84,629
	13	1	137,791
	14	1	67,937
	15	2	9,380; 12,440
T	10	12	5,313; 13,270; 26633 (*rpoB*); 42,947; 53,575; 55,547 (*atpB*); 64,816; 79,972 (*rpoA*); 81,903; 83,742; 115,516; 128,593
	11	8	3,834; 18,901 (*rpoC2*); 305,42; 125,407 (*rps15*); 128,239 (*ycf1*); 129,591 (*ycf1*); 130,221 (*ycf1*); 130,501 (*ycf1*)
	12	3	23,038; 36,512; 52,482
	13	2	10,782; 104,782
	14	1	59,954
	15	1	127,000 (*ycf1*)
C	10	1	22,958
G	12	1	73,448
AT	10	1	20,283 (*rpoC2*)
	12	2	68,297; 81,280
TA	10	1	127,419 (*ycf1*)
AATA	12	1	117,842 (*ndhD*)
ATAA	12	1	37,382
ATTT	12	1	68,040
TAGT	12	1	7,987
TTCT	16	1	65,070
GAAA	12	1	122,685
GAAT	12	1	6,966
TAAAT	15	1	79,640

a SSR-containing coding regions are indicated in parentheses.

At the genus- or tribe-level, the number of SSRs per plastome shows little variation: 59–63 in *Nicotiana* spp., 53–56 in *Solanum* spp., 51–53 in the *Guizotia*-*Helianthus*-*Parthenium* clade of Asteraceae [69]. The range is slightly larger in *Olea* (57–70), but the one with the fewest SSRs (*O*. *europaea* ssp. *cuspidata*) actually has seven mononucleotides just below the 10 bp cutoff, which are only one or two bp shorter than the counterparts in *O*. *europaea* ssp. *maroccana*. Among the *Cuscuta* species (29–49), the differences could be attributed to their different levels of plastome reduction. This is consistent with previous findings that SSR abundance is positively correlated with plastome size [70], [71]. In contrast with the conservation in the number of SSRs in closely related taxa, the number of SSRs differs considerably from one family to another. In the order Apiales, its range is 60–75 in Apiaceae, but only 37–46 in Araliaceae. In Lamiales, it is 57–70 in Oleaceae, but only 35 in *Sesamum* (Pedaliaceae) and 27 in *Boea* (Gesneriaceae). To determine if there are any shared SSRs in asterid plastomes, the SSR positions in the *A*. *polysticta* plastome were compared with those in *Helianthus annuus*, *Panax ginseng*, *Solanum lycopersicum*, *Boea hygrometrica*, *Olea europaea* cv. Bianchera and *Coffea arabica* plastomes. There is no SSR position common to all of these asterid plastomes. Two SSR positions are found in all but the *Helianthus* plastome. They are T homopolymers in *rpoC2* and *atpB*, corresponding to conserved lysine residues. Although SSRs in protein-coding regions tend to be conserved across lineages, they only represent a small portion of all plastome SSRs (14/57 in *A*. *polysticta*) and are unlikely to change in length due to the selection on maintaining reading frames. The higher evolutionary rates of noncoding regions create different sets of SSRs in different lineages that are more likely to be variable among haplotypes. This explains why the number of plastome SSRs changes dramatically from family to family and underscores the importance of a whole-plastome reference for SSR identification in related taxa.

### Long Repetitive Sequences

Ten sets of repetitive sequences that are 26 bp or longer were found in the *A*. *polysticta* plastome (Table 4; Figure 1). They were further divided into five categories based on the structure, including (1) tandem repeats, (2) dispersed direct repeats, (3) inverted repeats, (4) palindromic sequences, and (5) sequences that match their own reversed sequences. This five-type classification system is different from the seven-class system used by Timme et al. [30] in that we excluded SSRs (which are more abundant and were considered separately; see the previous section) and did not separate repeats in genic or intergenic regions into distinct categories (the numbers of long repeats were too few to warrant such detailed classification).

**Table 4:** Distribution of repetitive sequences in the *Ardisia polysticta* plastome.

No.^a^	Length (bp)	Type^b^	Start^c^ (gene position)	Repeat sequence	Region
1	42	D(I)	44,689 (*ycf3* intron 1); 100,403 (142141(I)) (*rps12*-*trnV*-*GAC*); 122,298 (*ndhA* intron)	YTACAGAACCGTACRTGAGATKTTCAYCTCATACGGCTCCTC	LSC; IR; SSC
2	36	T	93,339 (149,211) (*ycf2*); 93,375 (149,175) (*ycf2*)	TAGTGACGAYATTGATGCTAGTGACGAYATTGATGC	IR
3	35	D	39,754 (*psaB*); 41,978(*psaA*)	TGCAATAGCTAAATGATGRTGWGCAATATCRGTCA	LSC
4	30	R*	33,462 (*trnT*-*GGU*-*psbD*)	ATTATAWTATATATAATATATATWATATTA	LSC
5	30	P*	14,106 (*atpF*-*atpH*)	AAATATGAAAAATACGTATTTTTCATATTT	LSC
6	29	I	58,774 (*accD*); 58,814 (*accD*)	AATAATCACATTAATAGTTACATTGACAG	LSC
7	28	DI	8,839 (*trnS-GCU*); 36,668 (*trnS-UGA*)46,349 (*trnS-GGA*)	GGARAGAGAGGGATTCGAACCCTCGRTA	LSC
8	27	I	10,462 (*trnG*-*trnR*); 10,507 (*trnG*-*trnR*)	ATATATTCATTCTTTCTATTTCTTTCT	LSC
9	26	P*(D)	89,712 (152,848) (*ycf2*)	GAAGCAGATGATTAATCATCTGCTTC	IR
10	26	T	88,248 (154,312) (*trnI*-*CAU*-*ycf2*); 88,274 (154,286) (*trnI*-*CAU*-*ycf2*)	CTTTAGGAKAAATCAATGCAATTCAG	IR

a Repeats are numbered in the order of decreasing lengths.

b D: dispersed direct repeat; T: tandem repeat; I: inverted repeat; P*: palindromic sequence. R*: reversed sequence matches the original. Types indicated parenthetically are due to the inverted-repeat nature of the IR regions.

c For repeats in IR regions, the repeat sequences are shown for those in IRb with the start positions in IRa indicated in parentheses.

To investigate the evolution of these long repetitive sequences, we examined other asterids and outgroups (Table S1) for regions similar to the consensus sequences of the ten sets found in *A*. *polysticta*. Four sets of repetitive sequences were found to be conserved in asterids, *Spinacia*, and *Arabidopsis*: Nos. 1, 3, 7, and 9 (Table 4). The first three sets are found in all asterids except some parasitic taxa due to deletion or pseudogenization of certain genes (*ycf3*, *trnV*-*GAC*, *ndhA*, *psaA*, *psaB* and *trnS*-*GGA* in *Epifagus* [56], *ndhA* in *Cuscuta* spp. [4]). Two of these sets (Nos. 3 and 7) are similar portions of different photosystem I subunit genes (No. 3) or *trnS* genes (No. 7). Set No. 9 consists of a single palindromic sequence found in all asterids but *Cuscuta* spp., *Jasminum*, and *Trachelium*, probably because of high divergence levels of *ycf2* in these lineages [72]. Set No. 1 merits special attention because it has the longest consensus sequence (42 bp) among the ten sets and has been identified previously [30], [45], [67]. Additionally, this repeat was found in all four regions of asterid plastomes (i.e., LSC, SSC, IRa, and IRb).

In contrast to the conserved repeats, sets Nos. 2, 4, 6, 8 and 10 were absent in *Arabidopsis*, *Spinacia* and most asterids. Three of them (i.e., Nos. 4, 8, and 10) are located in intergenic regions and the other two in the fast evolving genes *accD* (No. 6) and *ycf2* (No. 2), which ranked as the third and the sixth most divergent genes in 17 tracheophyte plastomes [73]. The higher evolutionary rate explains why these repetitive sequences are more lineage-specific.

The remaining repeat (i.e., No. 5) has a more intriguing distribution across asterid lineages. It is absent in *Coffea*, *Boea*, *Sesamum*, Convolvulaceae and Apiaceae, but present in Oleaceae, Solanaceae, Araliaceae and four asteraceous genera. It consists of a perfect palindromic sequence in *A*. *polysticta*, but, in other asterids, it corresponds to a stretch of imperfect (except for *Solanum lycopersicum*) palindromic sequence capable of forming stem-loop structure (Table S3). Additionally, the loop sequence differs among genera and even among species in *Solanum*. As this sequence is generally found near the 3′ end of *atpH* and in the middle of a single transcription unit from *rps2* to *atpA* [61], it may play a role in the gene expression processes.

### Boundaries between Inverted Repeats and Single-copy Regions

**Figure 2:**
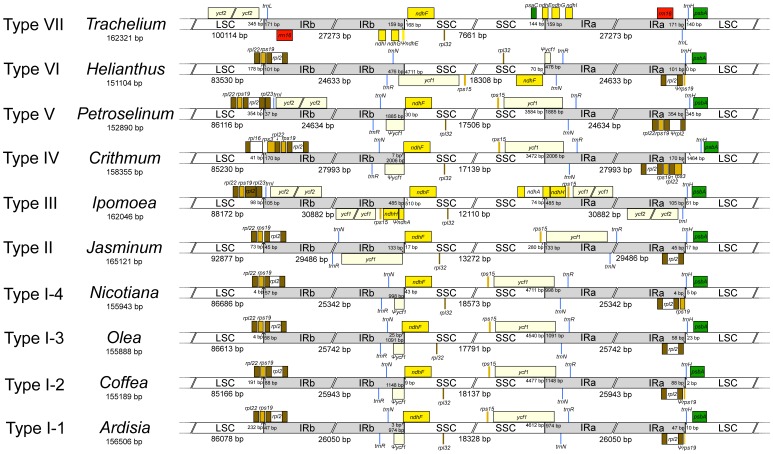
Comparison of boundaries between inverted repeats (IR) and single-copy (SC) regions in representative asterid plastomes. For a list of asterids found in different types, see Table S4.

To have a comprehensive overview of the asterid IR/SC boundary organizations, we compared the *A*. *polysticta* plastome with all available complete plastomes of nonparasitic euasterids. The asterid plastomes can be divided into seven types according to the extent of IR at the junctions between LSC and IRb (JLB), between SSC and IRb (JSB) and between SSC and IRa (JSA) (Figure 2; Table S4). Type I, represented by *A*. *polysticta*, has JLB within *rps19* or *rps19*-*rpl2*, JSA within *ndhF* or *trnN*-*GUU*-*ndhF*, and JSB within *ycf1*. This is the most common type in asterids, other eudicots [2] and the basal angiosperm *Amborella* [74]. Given its presence in the basal asterid *A*. *polysticta* and the outgroup *Spinacia*, this Type I organization probably represents the ancestral state of asterids. Because gene conversions occur constantly at the IR/SC boundaries, causing small expansions or contractions of IR even in within-genus comparison [75], Type I can be further divided into four subtypes. The subtyping is mainly based on whether JLB is within *rps19*-*rpl2* or *rps19* and whether JSB is within *trnN*-*GUU*-*ndhF* or *ndhF*. Among the subtypes, Type I-2, where JLB is within *rps19* and JSB *trnN*-*GUU*-*ndhF*, is the most common in euasterid plastomes and is found in all five euasterid orders (Table S4; Figure S1). If we consider this subtype as ancestral for euasterids, it could be inferred that several transitions have occurred from Type I-2 to other subtypes: Type I-3 in *Olea* spp., Type I-4 in a subclade of *Nicotiana*, and Type I-1 in *Boea*, *Hydrocotyle*, and *Solanum tuberosum* (Figure S1).

In comparison with Type I, other types of IR/SC organizations in asterids include those with substantial IR expansion (II, III, IV), IR contraction (III, V), or large rearrangements (VI, VII). The different types are also characterized by lengthening/shortening of IRs, LSC and SSC. For instance, the *Ipomoea* plastome (Type III) has a longer LSC and a shorter SSC, which are indicative of IR contraction at JLB and expansion at JSA, respectively. The events of IR expansion/contraction do not seem to have an apparent pattern in euasterids. Closely related taxa, such as *Petroselinum* and *Crithmum*, could change in the opposite directions at the same IR/SC boundary (Figure 2; Figure S1). In contrast to some seed plants [33], [76], [77], there seems to be no significant IR expansion/contraction event at JLA (junction between LSC and IRa) in the evolutionary history of the asterid plastomes.

### Phylogenetic Analysis of Asterid Plastomes

**Figure 3:**
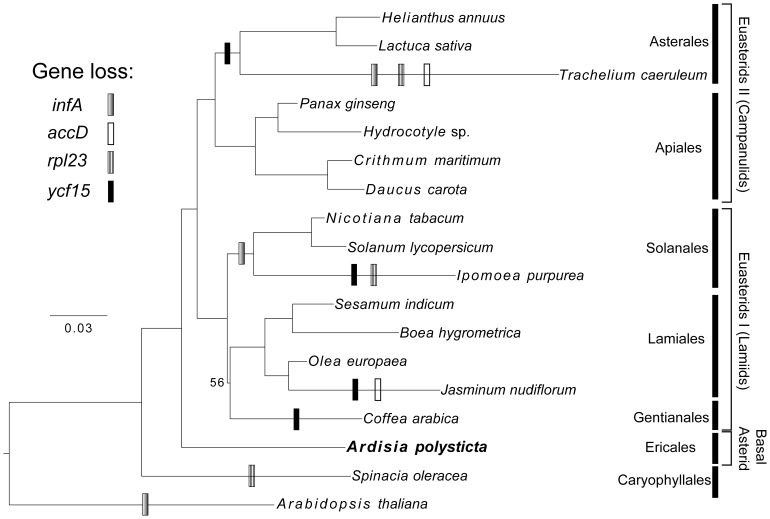
Maximum likelihood phylogeny of 78 plastome genes from 11 families (6 orders) of asterids. All nodes, except the one uniting Gentianales and Lamiales, received 100% bootstrap support. Gene loss events are mapped onto the tree in the most parsimonious way.

We reconstructed the phylogenetic relationships of representative asterid taxa and two outgroups using 78 orthologous genes shared by all plastomes. The phylogeny shown in Figure 3 contains 16 ingroups that best represent asterid diversity (see Materials and Methods for the over-representation issue of certain genera). Additionally, a more comprehensive sampling that includes all usable nonparasitic euasterid plastomes is shown in Figure S1. In terms of order- or family-level diversity, these are by far the most comprehensive phylogenetic analyses of asterids based on complete plastomes. The topology was not affected by the taxon sampling. Both maximum likelihood trees strongly support the basal position of Ericales within asterids and the subdivision of euasterids into euasterids I (Gentianales, Lamiales, Solanales) and euasterids II (Apiales, Asterales).

It should be noted that all the nodes in the whole-plastome tree of 16 asterids received 100% support in the ML bootstrap analyses, except for the one grouping Gentianales and Lamiales (Figure 3). In the latest APG classification system, Lamiales is more closely related to Solanales than either is to Gentianales [3], which is consistent with a phylogeny of 111 taxa based on three plasid protein-coding genes and three plastid non-coding regions [6]. However, many other phylogenetic analyses with more extensive taxon sampling and/or based on more molecular markers resulted in topologies different from that of the APG III system. In the evolutionary tree based on four mitochondrial genes, the relationships among the major euasterids I orders are largely unresolved [78]. On the contrary, Gentianales is the sister group to Solanales in the phylogenetic trees inferred from 77 nuclear genes [79], a combination of one nuclear and three plastid genes [80], or 17 regions from all three genomes [81]. Phylogenetic trees based on whole-plastome data show a sister relationship of Gentianales either with Solanales [82] or with Lamiales [45], [83]. As these three studies used almost all protein-coding and rRNA genes in an angiosperm plastome, the discrepancy is best explained by the differences in taxon sampling. Whereas the parasitic taxa *Epifagus* and *Cuscuta* were included in Moore et al. [82], Jansen et al. [83] and Yi and Kim [45] included multiple plastomes from a single genus (*Olea*, *Nicotiana*, *Solanum*) or even a single species (*Olea europaea*) in their analyses. Our exclusion of the parasitic lineages, which are likely to cause long-branch attraction problem due to their accelerated evolutionary rates, resulted in the grouping of Gentianales and Lamiales (Figures 3 and S1). Although the control for reducing overrepresentations of certain taxa improves the bootstrap value of this clade (56 vs. 49; cf. Figures 3 and S1), the support is still relatively weak. Examination of the bootstrap trees showed that the clustering of Gentianales and Solanales represents the best supported alternative. This result indicates that the present dataset could not resolve the relationships within euasterids I. Further analyses with complete plastome sequences from other euasterids I and more extensive taxon sampling within Gentianales would be needed to resolve the relationships.

### Gene Content Evolution

When compared with the gene content of *A*. *polysticta* plastome, six protein-coding genes have been differentially lost in asterid lineages (Table 5). Three of these that have been pseudogenized or deleted outside of the asterids are: *infA* (lost in many rosids and other angiosperm lineages [84]), *rpl23* (*Spinacia* [85] and several gymnosperms [33]), and *accD* (many Poales families [86]). Both *accD* and *rpl23* were found to be essential for *Nicotiana* [87], [88] and *rpl23* was suggested to be replaced by a nuclear homologue in *Spinacia* [85]. The *infA* gene was found to have been independently transferred to and expressed in the nucleus with a transit peptide in lineages without intact plastome *infA* (including *Solanum lycopersicum*) [84]. These suggest that their loss in different asterid lineages might indicate independent functional replacement by a nuclear copy.

**Table 5:** Loss and gain of plastome protein-coding genes relative to *Ardisia polysticta* in nonparasitic euasterids.

Taxa^a^	Loss (−) and gain (+)^b^
*Trachelium*	− *ycf15*, *clpP*, *rpl23*, *infA*, *accD*, *ndhK*+ two *psbJ* duplicates
*Ipomoea*	− *ycf15*, *infA*, *rpl23* c
*Jasminum*	− *ycf15*, *accD*
*Ageratina*, *Anthriscus*, *Coffea*, *Guizotia*, *Helianthus*, *Jacobaea*, *Lactuca*, *Oxypolis*	− *ycf15*
*Atropa*, *Capsicum*, *Datura*, *Nicotiana* spp., *Solanum* spp.	− *infA*
*Boea*, *Coffea*, *Crithmum*, *Daucus*, *Eleutherococcus*, *Hydrocotyle*, *Olea* spp., *Panax*, *Petroselinum*, *Sesamum*	None

a *Parthenium argentatum* (Asteraceae) is not included. For reasons, see Materials and Methods.

b All losses and gains were manually verified by BLAST searches. Differences in IR duplicates are not included.

c Pseudogenization evidenced by an extended 3′ end, two frameshift mutations and an accelerated evolutionary rate (McNeal et al., 2007).

Mapping of gene loss events onto the plastome phylogeny (Figure 3; Figure S1) shows that many losses occurred in lineages with plastomes characterized by large inversions (*Trachelium* [72], *Jasminum* [89]) or IR contraction/expansion (*Ipomoea*, *Jaminum*; Figure 2; Table S4). Specifically, the *Trachelium* plastome, which has been extensively rearranged [72], has lost all the six genes (Table 5). Moreover, these lineages have longer branches compared to their sister groups (Figure 3; Figure S1) and they have the smallest SSC and the largest LSC among asterids (Figure 2). This is intriguing because the opposite would be expected due to higher evolutionary rate of genes in SSC than in LSC [45], [46]. More studies are needed to investigate such association of higher evolutionary rate, changes in plastome organization, and gene loss across a larger range of angiosperm lineages.

### Evolution of *ycf15*

The hypothetical gene *ycf15* has been lost six times in the asterid phylogeny (Figure S1). Based on nucleotide sequence similarity, two regions separated by an intervening sequence of 250–300 bp in plastomes of several basal angiosperms, monocots, and non-asterid eudicots correspond to the 5′ (1–154) and 3′ (155–264) portions of the *Nicotiana ycf15* [70], [90]. However, the intervening sequence was shown to be absent in a few asterids, including *Epifagus virginiana*, *Cuscuta reflexa*, *Panax ginseng*, and two other solanaceous genera *Atropa* and *Solanum* [70], [90]. In this study, we further confirmed the absence of the intervening sequence in other complete asterid plastomes, including those from Apiaceae, Lamiales, and the basal asterid *Ardisia*, thus pinpointing the time of its loss to the range after the divergence of Caryophyllales and before the Ericales-euasterids split. Amplification of *ycf15* from *Spinacia* cDNA showed that it was transcribed, but the intervening sequence was not removed in the RNA transcript [90]. Since premature stop codons in the intervening sequence would result in truncated protein products without the region homologous to the *Nicotiana ycf15* 3′ portion, this led to the suggestion that *ycf15* was probably not a protein-coding gene [70], [90].

However, even in asterid plastomes where a continuous region homologous to the *Nicotiana ycf15* occurs, frameshift indels are found in the *ycf15* 3′ portion of non-Solanaceae asterids, resulting in premature stop codons in Lamiales and *Ardisia* or an extended but dissimilar 3′ portion in *Eleutherococcus* and *Panax* (Figure S1; Figure S2). The high length and sequence variability of the 3′ portion suggests that it plays a minor role in the function of *ycf15*. Compared to the 3′ portion, the 5′ portion is largely invariable and no frameshift mutation has been observed based on available plastome sequences. Alignment of the amino acid sequences also shows that there is a conserved *ycf15* region that corresponds to the central region of *ycf15* in asterids and the 5′ half in non-asterids (Figure S2). If *ycf15* is indeed expressed as polypeptides, this region would probably assume the main functional role.

### Conclusions

The complete plastome sequence of the basal asterid *Ardisia polysticta* was obtained using Illumina technology and Sanger sequencing. The *Ardisia* plastome has the gene content and organization typical of asterids and most angiosperms. By comparing the divergence levels of intergenic regions between *Ardisia* and euasterids, we found candidate regions of potential phylogenetic utility for this speciose genus. Using the *Ardisia* plastome as a reference sheds light on the characteristics and diversity of asterid plastomes with respect to GC content, plastome organization, gene content and content of repetitive sequences. Phylogenetic analysis based on complete plastomes highlights uncertainty in the position of Gentianales within euasterids I, which merits further studies.

## Supporting Information

Figure S1 **Maximum likelihood phylogeny of 78 plastome genes from 35 nonparasitic asterids (11 families, 6 orders).** All nodes, except where noted, received 100% bootstrap support. Gene loss events are mapped onto the tree in the most parsimonious way (Table 5). Types of inverted repeat/single copy boundary organization are also indicated (Table S4). All euasterid taxa, except where noted, have Type I-2 plastomes. Indel events within the 3′ portion of *ycf15* (Figure S2B) are also mapped onto the tree.
(TIF)

Figure S2 **Alignment of** ***ycf15*** **.** A. Alignment of *ycf15* amino acid sequences in *Calycanthus*, *Arabidopsis*, *Spinacia*, and asterids. The arrow indicates the divide between the 5′ and 3′ portions of *ycf15* in *Nicotiana tabacum*, to which the homologous regions are separated by a 250–300 bp intervening sequence in non-asterid angiosperms. B. Alignment of asterid *ycf15* nucleotide sequences corresponding the boxed region of *Nicotiana tabacum* in A. In-frame stop codons are boxed. Arrows indicate the six non-triplet indels.
(TIF)

Table S1 Accession numbers of complete plastome sequences of asterids and of those included in the phylogenetic tree in Figure 3 (bold).
(DOCX)

Table S2 Genes encoded in the *Ardisia polysticta* plastome.
(DOCX)

Table S3 Sequences that correspond to the palindromic sequence in *atpF*-*atpH* in *A*. *polysticta* plastome (No. 5 in Table 4).
(DOCX)

Table S4 Types of plastome Inverted Repeat/Single Copy boundaries in asterids.
(DOCX)

## References

[1] Rodríguez-Ezpeleta N, Brinkmann H, Burey SC, Roure B, Burger G, et al (2005) Monophyly of primary photosynthetic eukaryotes: green plants, red algae, and glaucophytes. Curr Biol 15: 1325–1330. 16051178 10.1016/j.cub.2005.06.040

[2] Ravi V, Khurana JP, Tyagi AK, Khurana P (2008) An update on chloroplast genomes. Plant Syst Evol 271: 101–122.

[3] APG III (2009) An update of the Angiosperm Phylogeny Group classification for the orders and families of flowering plants: APG III. Bot J Linn Soc 161: 105–121.

[4] McNeal JR, Kuehl JV, Boore JL, dePamphilis CW (2007) Complete plastid genome sequences suggest strong selection for retention of photosynthetic genes in the parasitic plant genus *Cuscuta* . BMC Plant Biol 7: 57. 17956636 10.1186/1471-2229-7-57 PMC2216012

[5] Wolfe KH, Morden CW, Ems SC, Palmer JD (1992) Rapid evolution of the plastid translational apparatus in a nonphotosynthetic plant: loss or accelerated sequence evolution of tRNA and ribosomal protein genes. J Mol Evol 35: 304–317. 1404416 10.1007/BF00161168

[6] Bremer B, Bremer K, Heidari N, Erixon P, Olmstead RG, et al (2002) Phylogenetics of asterids based on 3 coding and 3 non-coding chloroplast DNA markers and the utility of non-coding DNA at higher taxonomic levels. Mol Phylogen Evol 24: 274–301. 10.1016/s1055-7903(02)00240-3 12144762

[7] Ståhl B, Anderberg AA (2004) Myrisinaceae. In: Kubitzki K, editor. The Families and Genera of Vascular Plants. Berlin: Springer-Verlag. 266–281.

[8] Chen J, Pipoly JJ (1996) Myrisnaceae. In: Wu Z, Raven PH, editors. Flora of China. 1 ed. Beijing: Science Press. 1–38.

[9] Grierson AJC, Long DG (1999) Flora of Bhutan. Edinburgh: Royal Botanic Garden.

[10] Sundriyal M, Sundriyal RC (2001) Wild edible plants of the Sikkim Himalaya: Nutritive values of selected species. Econ Bot 55: 377–390.

[11] Chen C (1979) Angiospermae, Dicotyledoneae, Mysinaceae. In: Chen C, editor. Flora Reipublicae Popularis Sinicae Tomus 58. Beijing: Science Press. 1–147.

[12] Ramírez-Mares MV, González de Mejía E (2003) Comparative study of the antioxidant effect of ardisin and epigallocatechin gallate in rat hepatocytes exposed to benomyl and 1-nitropyrene. Food Chem Toxicol 41: 1527–1535. 12963005 10.1016/s0278-6915(03)00169-8

[13] Piacente S, Pizza C, De Tommasi N, Mahmood N (1996) Constituents of *Ardisia japonica* and their *in vitro* anti-HIV activity. J Nat Prod 59: 565–569. 8786362 10.1021/np960074h

[14] González de Mejía E, Chandra S, Ramírez-Mares M, Wang W (2006) Catalytic inhibition of human DNA topoisomerase by phenolic compounds in *Ardisia compressa* extracts and their effect on human colon cancer cells. Food Chem Toxicol 44: 1191–1203. 16540225 10.1016/j.fct.2006.01.015

[15] Jia Z, Koike K, Ohmoto T, Ni M (1994) Triterpenoid saponins from *Ardisia crenata* . Phytochemistry 37: 1389–1396. 7765756 10.1016/s0031-9422(00)90418-7

[16] Yang LK, Khoo-Beattie C, Goh KL, Chng BL, Yoganathan K, et al (2001) Ardisiaquinones from *Ardisia teysmanniana* . Phytochemistry 58: 1235–1238. 11738414 10.1016/s0031-9422(01)00317-x

[17] Sumino M, Sekine T, Ruangrungsi N, Igarashi K, Ikegami F (2002) Ardisiphenols and other antioxidant principles from the fruits of *Ardisia colorata* . Chem Pharm Bull 50: 1484–1487. 12419914 10.1248/cpb.50.1484

[18] Kobayashi H, de Mejia E (2005) The genus *Ardisia*: a novel source of health-promoting compounds and phytopharmaceuticals. J Ethnopharmacol 96: 347–354. 15619551 10.1016/j.jep.2004.09.037

[19] Sims J (1818) Curtis's Botanical Magazine Vol. 45. London: Sherwood, Neely, & Jones.

[20] Niu H-Y, Hong L, Wang Z-F, Shen H, Ye W-H, et al (2012) Inferring the invasion history of coral berry *Ardisia crenata* from China to the USA using molecular markers. Ecol Res 27: 809–818.

[21] Lemaire B, Smets E, Dessein S (2011) Bacterial leaf symbiosis in *Ardisia* (Myrsinoideae, Primulaceae): molecular evidence for host specificity. Res Microbiol 162: 528–534. 21527340 10.1016/j.resmic.2011.04.003

[22] Hu CM (1999) New synonyms and combinations in Asiatic *Ardisia* (Myrsinaceae). Blumea 44: 391–406.

[23] Yang Y-P (1999) An enumeration of Myrsinaceae of Taiwan. Bot Bull Acad Sin 40: 39–47.

[24] Gould SB, Waller RR, McFadden GI (2008) Plastid evolution. Annu Rev Plant Biol 59: 491–517. 18315522 10.1146/annurev.arplant.59.032607.092915

[25] Podolak I, Galanty A, Sobolewska D (2010) Saponins as cytotoxic agents: a review. Phytochem Rev 9: 425–474. 20835386 10.1007/s11101-010-9183-z PMC2928447

[26] Kesselmeier J (1980) Development of chloro-etioplasts containing prolamellar bodies and steroidal saponins in suspension cultures of *Nicotiana tabacum* . Protoplasma 104: 295–306.

[27] Kumar S, Daniell H (2004) Engineering the chloroplast genome for hyperexpression of human therapeutic proteins and vaccine antigens. In: Balbás P, Lorence A, editors. Recombinant Gene Expression: Humana Press. 365–383. 10.1385/1-59259-774-2:365 15269437

[28] Xu L-l, Li T-j, Zhang M-y, Yi G-m, Liao L (2009) Interspecific relationships and variation of 12 species in *Ardisia* Sw. (Myrsinaceae) based on ITS and trnL-F data sets. Acta Hortic Sin 36: 1531–1537.

[29] Clegg MT, Gaut BS, Learn GH, Morton BR (1994) Rates and patterns of chloroplast DNA evolution. Proc Natl Acad Sci USA 91: 6795–6801. 8041699 10.1073/pnas.91.15.6795 PMC44285

[30] Timme RE, Kuehl JV, Boore JL, Jansen RK (2007) A comparative analysis of the *Lactuca* and *Helianthus* (Asteraceae) plastid genomes: identification of divergent regions and categorization of shared repeats. Am J Bot 94: 302–312. 21636403 10.3732/ajb.94.3.302

[31] Provan J, Powell W, Hollingsworth PM (2001) Chloroplast microsatellites: new tools for studies in plant ecology and evolution. Trends Ecol Evol 16: 142–147. 11179578 10.1016/s0169-5347(00)02097-8

[32] Luo C, Tsementzi D, Kyrpides N, Read T, Konstantinidis KT (2012) Direct comparisons of Illumina vs. Roche 454 sequencing technologies on the same microbial community DNA sample. PLoS One 7: e30087. 22347999 10.1371/journal.pone.0030087 PMC3277595

[33] Lin C-P, Wu C-S, Huang Y-Y, Chaw S-M (2012) The complete chloroplast genome of *Ginkgo biloba* reveals the mechanism of inverted repeat contraction. Genome Biol Evol 4: 374–381. 22403032 10.1093/gbe/evs021 PMC3318433

[34] Kuang D-Y, Wu H, Wang Y-L, Gao L-M, Zhang S-Z, et al (2011) Complete chloroplast genome sequence of *Magnolia kwangsiensis* (Magnoliaceae): implication for DNA barcoding and population genetics. Genome 54: 663–673. 21793699 10.1139/g11-026

[35] Zerbino DR, Birney E (2008) Velvet: Algorithms for de novo short read assembly using de Bruijn graphs. Genome Res 18: 821–829. 18349386 10.1101/gr.074492.107 PMC2336801

[36] Altschul SF, Madden TL, Schäffer AA, Zhang J, Zhang Z, et al (1997) Gapped BLAST and PSI-BLAST: a new generation of protein database search programs. Nucleic Acids Res 25: 3389–3402. 9254694 10.1093/nar/25.17.3389 PMC146917

[37] Camacho C, Coulouris G, Avagyan V, Ma N, Papadopoulos J, et al (2009) BLAST+: architecture and applications. BMC Bioinformatics 10: 421. 20003500 10.1186/1471-2105-10-421 PMC2803857

[38] Benson DA, Karsch-Mizrachi I, Clark K, Lipman DJ, Ostell J, et al (2012) GenBank. Nucleic Acids Res 40: D48–D53. 22144687 10.1093/nar/gkr1202 PMC3245039

[39] Li H, Durbin R (2009) Fast and accurate short read alignment with Burrows-Wheeler transform. Bioinformatics 25: 1754–1760. 19451168 10.1093/bioinformatics/btp324 PMC2705234

[40] Robinson JT, Thorvaldsdottir H, Winckler W, Guttman M, Lander ES, et al (2011) Integrative genomics viewer. Nat Biotechnol 29: 24–26. 21221095 10.1038/nbt.1754 PMC3346182

[41] Wyman SK, Jansen RK, Boore JL (2004) Automatic annotation of organellar genomes with DOGMA. Bioinformatics 20: 3252–3255. 15180927 10.1093/bioinformatics/bth352

[42] Lowe TM, Eddy SR (1997) tRNAscan-SE: a program for improved detection of transfer RNA genes in genomic sequence. Nucleic Acids Res 25: 955–964. 9023104 10.1093/nar/25.5.955 PMC146525

[43] Lohse M, Drechsel O, Bock R (2007) OrganellarGenomeDRAW (OGDRAW): a tool for the easy generation of high-quality custom graphical maps of plastid and mitochondrial genomes. Curr Genet 52: 267–274. 17957369 10.1007/s00294-007-0161-y

[44] Conant GC, Wolfe KH (2008) GenomeVx: simple web-based creation of editable circular chromosome maps. Bioinformatics 24: 861–862. 18227121 10.1093/bioinformatics/btm598

[45] Yi D-K, Kim K-J (2012) Complete chloroplast genome sequences of important oilseed crop *Sesamum indicum* . PLoS One 7: e35872. 22606240 10.1371/journal.pone.0035872 PMC3351433

[46] Yi D-K, Lee H-L, Sun B-Y, Chung M, Kim K-J (2012) The complete chloroplast DNA sequence of *Eleutherococcus senticosus* (Araliaceae); Comparative evolutionary analyses with other three asterids. Molecules and Cells 33: 497–508. 22555800 10.1007/s10059-012-2281-6 PMC3887725

[47] Edgar RC (2004) MUSCLE: multiple sequence alignment with high accuracy and high throughput. Nucleic Acids Res 32: 1792–1797. 15034147 10.1093/nar/gkh340 PMC390337

[48] Felsenstein J (1989) PHYLIP - Phylogeny Inference Package (Version 3.2). Cladistics 5: 164–166.

[49] Thurston MI, Field D (2005) Msatfinder: detection and characterisation of microsatellites. Distributed by the authors.

[50] Kurtz S, Schleiermacher C (1999) REPuter: fast computation of maximal repeats in complete genomes. Bioinformatics 15: 426–427. 10366664 10.1093/bioinformatics/15.5.426

[51] Kumar S, Hahn F, McMahan C, Cornish K, Whalen M (2009) Comparative analysis of the complete sequence of the plastid genome of *Parthenium argentatum* and identification of DNA barcodes to differentiate *Parthenium* species and lines. BMC Plant Biol 9: 131. 19917140 10.1186/1471-2229-9-131 PMC2784773

[52] Li L, Stoeckert CJ, Roos DS (2003) OrthoMCL: Identification of ortholog groups for eukaryotic genomes. Genome Res 13: 2178–2189. 12952885 10.1101/gr.1224503 PMC403725

[53] Guindon S, Gascuel O (2003) A simple, fast, and accurate algorithm to estimate large phylogenies by maximum likelihood. Syst Biol 52: 696–704. 14530136 10.1080/10635150390235520

[54] Felsenstein J (1985) Confidence limits on phylogenies: an approach using the bootstrap. Evolution 39: 783–791. 28561359 10.1111/j.1558-5646.1985.tb00420.x

[55] Hildebrand M, Hallick RB, Passavant CW, Bourque DP (1988) Trans-splicing in chloroplasts: the *rps*12 loci of *Nicotiana tabacum* . Proc Natl Acad Sci USA 85: 372–376. 3422433 10.1073/pnas.85.2.372 PMC279550

[56] Wolfe KH, Morden CW, Palmer JD (1992) Function and evolution of a minimal plastid genome from a nonphotosynthetic parasitic plant. Proc Natl Acad Sci USA 89: 10648–10652. 1332054 10.1073/pnas.89.22.10648 PMC50398

[57] Lockhart PJ, Steel MA, Hendy MD, Penny D (1994) Recovering evolutionary trees under a more realistic model of sequence evolution. Mol Biol Evol 11: 605–612. 19391266 10.1093/oxfordjournals.molbev.a040136

[58] Lind PA, Andersson DI (2008) Whole-genome mutational biases in bacteria. Proc Natl Acad Sci USA 105: 17878–17883. 19001264 10.1073/pnas.0804445105 PMC2584707

[59] Perry AS, Wolfe KH (2002) Nucleotide substitution rates in legume chloroplast DNA depend on the presence of the inverted repeat. J Mol Evol 55: 501–508. 12399924 10.1007/s00239-002-2333-y

[60] Shinozaki K, Hayashida N, Sugiura M (1988) *Nicotiana* chloroplast genes for components of the photosynthetic apparatus. Photosynthesis Res 18: 7–31. 10.1007/BF00042978 24425159

[61] Kanno A, Hirai A (1993) A transcription map of the chloroplast genome from rice (*Oryza sativa*). Curr Genet 23: 166–174. 8381719 10.1007/BF00352017

[62] Yesson C, Toomey NH, Culham A (2009) Cyclamen: time, sea and speciation biogeography using a temporally calibrated phylogeny. J Biogeogr 36: 1234–1252.

[63] Mráz P, Garcia-Jacas N, Gex-Fabry E, Susanna A, Barres L, et al (2012) Allopolyploid origin of highly invasive *Centaurea stoebe* s.l. (Asteraceae). Mol Phylogen Evol 62: 612–623. 10.1016/j.ympev.2011.11.006 22126902

[64] Ge XJ, Chiang YC, Chou CH, Chiang TY (2002) Nested clade analysis of *Dunnia sinensis* (Rubiaceae), a monotypic genus from China based on organelle DNA sequences. Conserv Genet 3: 351–362.

[65] Huang C-C, Hung K-H, Hwang C-C, Huang J-C, Lin H-D, et al (2011) Genetic population structure of the alpine species *Rhododendron pseudochrysanthum sensu lato* (Ericaceae) inferred from chloroplast and nuclear DNA. BMC Evol Biol 11: 108. 21501530 10.1186/1471-2148-11-108 PMC3096940

[66] Shaw J, Lickey EB, Beck JT, Farmer SB, Liu W, et al (2005) The tortoise and the hare II: relative utility of 21 noncoding chloroplast DNA sequences for phylogenetic analysis. Am J Bot 92: 142–166. 21652394 10.3732/ajb.92.1.142

[67] Mariotti R, Cultrera N, Diez C, Baldoni L, Rubini A (2010) Identification of new polymorphic regions and differentiation of cultivated olives (*Olea europaea* L.) through plastome sequence comparison. BMC Plant Biol 10: 211. 20868482 10.1186/1471-2229-10-211 PMC2956560

[68] Shaw J, Lickey EB, Schilling EE, Small RL (2007) Comparison of whole chloroplast genome sequences to choose noncoding regions for phylogenetic studies in angiosperms: the tortoise and the hare III. Am J Bot 94: 275–288. 21636401 10.3732/ajb.94.3.275

[69] Nie X, Lv S, Zhang Y, Du X, Wang L, et al (2012) Complete chloroplast genome sequence of a major invasive species, crofton weed (*Ageratina adenophora*). PLoS One 7: e36869. 22606302 10.1371/journal.pone.0036869 PMC3350484

[70] Raubeson L, Peery R, Chumley T, Dziubek C, Fourcade HM, et al (2007) Comparative chloroplast genomics: analyses including new sequences from the angiosperms *Nuphar advena* and *Ranunculus macranthus* . BMC Genomics 8: 174. 17573971 10.1186/1471-2164-8-174 PMC1925096

[71] Huotari T, Korpelainen H (2012) Complete chloroplast genome sequence of *Elodea canadensis* and comparative analyses with other monocot plastid genomes. Gene 508: 96–105. 22841789 10.1016/j.gene.2012.07.020

[72] Haberle R, Fourcade HM, Boore J, Jansen R (2008) Extensive rearrangements in the chloroplast genome of *Trachelium caeruleum* are associated with repeats and tRNA genes. J Mol Evol 66: 350–361. 18330485 10.1007/s00239-008-9086-4

[73] Kim KJ, Lee HL (2004) Complete chloroplast genome sequences from Korean ginseng (*Panax schinseng* Nees) and comparative analysis of sequence evolution among 17 vascular plants. DNA Res 11: 247–261. 15500250 10.1093/dnares/11.4.247

[74] Goremykin VV, Hirsch-Ernst KI, Wölfl S, Hellwig FH (2003) Analysis of the *Amborella trichopoda* chloroplast genome sequence suggests that *Amborella* is not a basal angiosperm. Mol Biol Evol 20: 1499–1505. 12832641 10.1093/molbev/msg159

[75] Goulding SE, Wolfe KH, Olmstead RG, Morden CW (1996) Ebb and flow of the chloroplast inverted repeat. Mol Gen Genet 252: 195–206. 8804393 10.1007/BF02173220

[76] Wu C-S, Wang Y-N, Liu S-M, Chaw S-M (2007) Chloroplast genome (cpDNA) of *Cycas taitungensis* and 56 cp protein-coding genes of *Gnetum parvifolium*: insights into cpDNA evolution and phylogeny of extant seed plants. Mol Biol Evol 24: 1366–1379. 17383970 10.1093/molbev/msm059

[77] Chang C-C, Lin H-C, Lin IP, Chow T-Y, Chen H-H, et al (2006) The chloroplast genome of *Phalaenopsis aphrodite* (Orchidaceae): comparative analysis of evolutionary rate with that of grasses and its phylogenetic implications. Mol Biol Evol 23: 279–291. 16207935 10.1093/molbev/msj029

[78] Qiu Y-L, Li L, Wang B, Xue J-Y, Hendry TA, et al (2010) Angiosperm phylogeny inferred from sequences of four mitochondrial genes. J Syst Evol 48: 391–425.

[79] Finet C, Timme RE, Delwiche CF, Marlétaz F (2010) Multigene phylogeny of the green lineage reveals the origin and diversification of land plants. Curr Biol 20: 2217–2222. 21145743 10.1016/j.cub.2010.11.035

[80] Albach DC, Soltis PS, Soltis DE, Olmstead RG (2001) Phylogenetic analysis of asterids based on sequences of four genes. Ann Mo Bot Gard 88: 163–212.

[81] Soltis DE, Smith SA, Cellinese N, Wurdack KJ, Tank DC, et al (2011) Angiosperm phylogeny: 17 genes, 640 taxa. Am J Bot 98: 704–730. 21613169 10.3732/ajb.1000404

[82] Moore MJ, Soltis PS, Bell CD, Burleigh JG, Soltis DE (2010) Phylogenetic analysis of 83 plastid genes further resolves the early diversification of eudicots. Proc Natl Acad Sci USA 107: 4623–4628. 20176954 10.1073/pnas.0907801107 PMC2842043

[83] Jansen RK, Cai Z, Raubeson LA, Daniell H, dePamphilis CW, et al (2007) Analysis of 81 genes from 64 plastid genomes resolves relationships in angiosperms and identifies genome-scale evolutionary patterns. Proc Natl Acad Sci USA 104: 19369–19374. 18048330 10.1073/pnas.0709121104 PMC2148296

[84] Millen RS, Olmstead RG, Adams KL, Palmer JD, Lao NT, et al (2001) Many parallel losses of *infA* from chloroplast DNA during angiosperm evolution with multiple independent transfers to the nucleus. Plant Cell 13: 645–658. 11251102 10.1105/tpc.13.3.645 PMC135507

[85] Bubunenko MG, Schmidt J, Subramanian AR (1994) Protein substitution in chloroplast ribosome evolution: a eukaryotic cytosolic protein has replaced its organelle homologue (L23) in spinach. J Mol Biol 240: 28–41. 8021938 10.1006/jmbi.1994.1415

[86] Harris M, Meyer G, Vandergon T, Vandergon V (2013) Loss of the acetyl-CoA carboxylase (*accD*) gene in Poales. Plant Mol Biol Report 31: 21–31.

[87] Kode V, Mudd EA, Iamtham S, Day A (2005) The tobacco plastid *accD* gene is essential and is required for leaf development. Plant J 44: 237–244. 16212603 10.1111/j.1365-313X.2005.02533.x

[88] Fleischmann TT, Scharff LB, Alkatib S, Hasdorf S, Schöttler MA, et al (2011) Nonessential plastid-encoded ribosomal proteins in tobacco: a developmental role for plastid translation and implications for reductive genome evolution. Plant Cell 23: 3137–3155. 21934145 10.1105/tpc.111.088906 PMC3203423

[89] Lee H-L, Jansen RK, Chumley TW, Kim K-J (2007) Gene relocations within chloroplast genomes of *Jasminum* and *Menodora* (Oleaceae) are due to multiple, overlapping inversions. Mol Biol Evol 24: 1161–1180. 17329229 10.1093/molbev/msm036

[90] Schmitz-Linneweber C, Maier RM, Alcaraz JP, Cottet A, Herrmann RG, et al (2001) The plastid chromosome of spinach (*Spinacia oleracea*): complete nucleotide sequence and gene organization. Plant Mol Biol 45: 307–315. 11292076 10.1023/a:1006478403810

