# Supplementary figures and images for: Complete Plastid Genome Sequence of the Basal Asterid Ardisia polysticta Miq. and Comparative Analyses of Asterid Plastid Genomes
Source: PLoS One. 2013 Apr 30;8(4):e62548. doi: 10.1371/journal.pone.0062548 (PMC3640096; doi:10.1371/journal.pone.0062548)

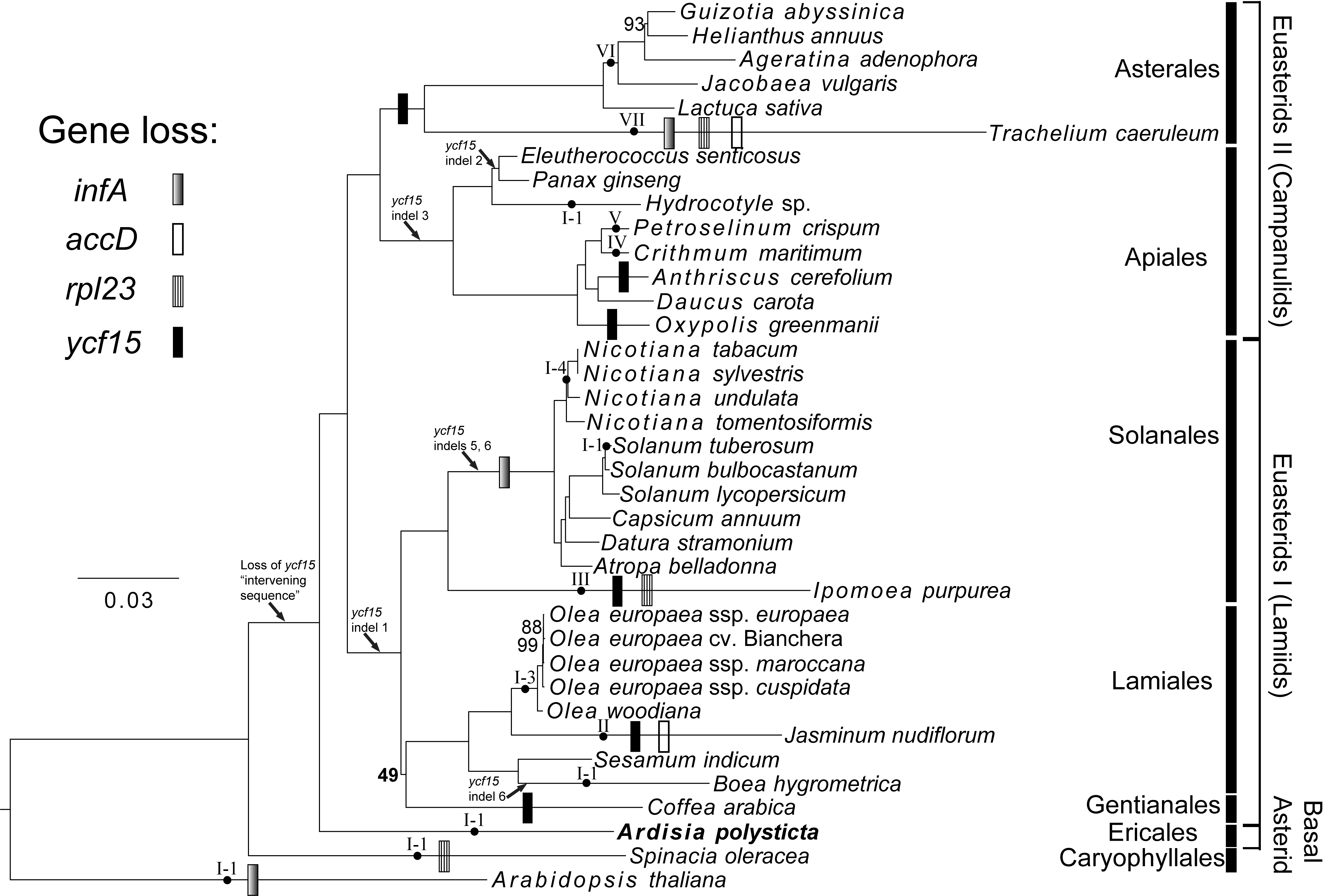

Supplement: Figure S1 — Maximum likelihood phylogeny of 78 plastome genes from 35 nonparasitic asterids (11 families, 6 orders). All nodes, except where noted, received 100% bootstrap support. Gene loss events are mapped onto the tree in the most parsimonious way (Table 5). Types of inverted repeat/single copy boundary organization are also indicated (Table S4). All euasterid taxa, except where noted, have Type I-2 plastomes. Indel events within the 3′ portion of ycf15 (Figure S2B) are also mapped onto the tree. (TIF) [file pone.0062548.s001.tif]

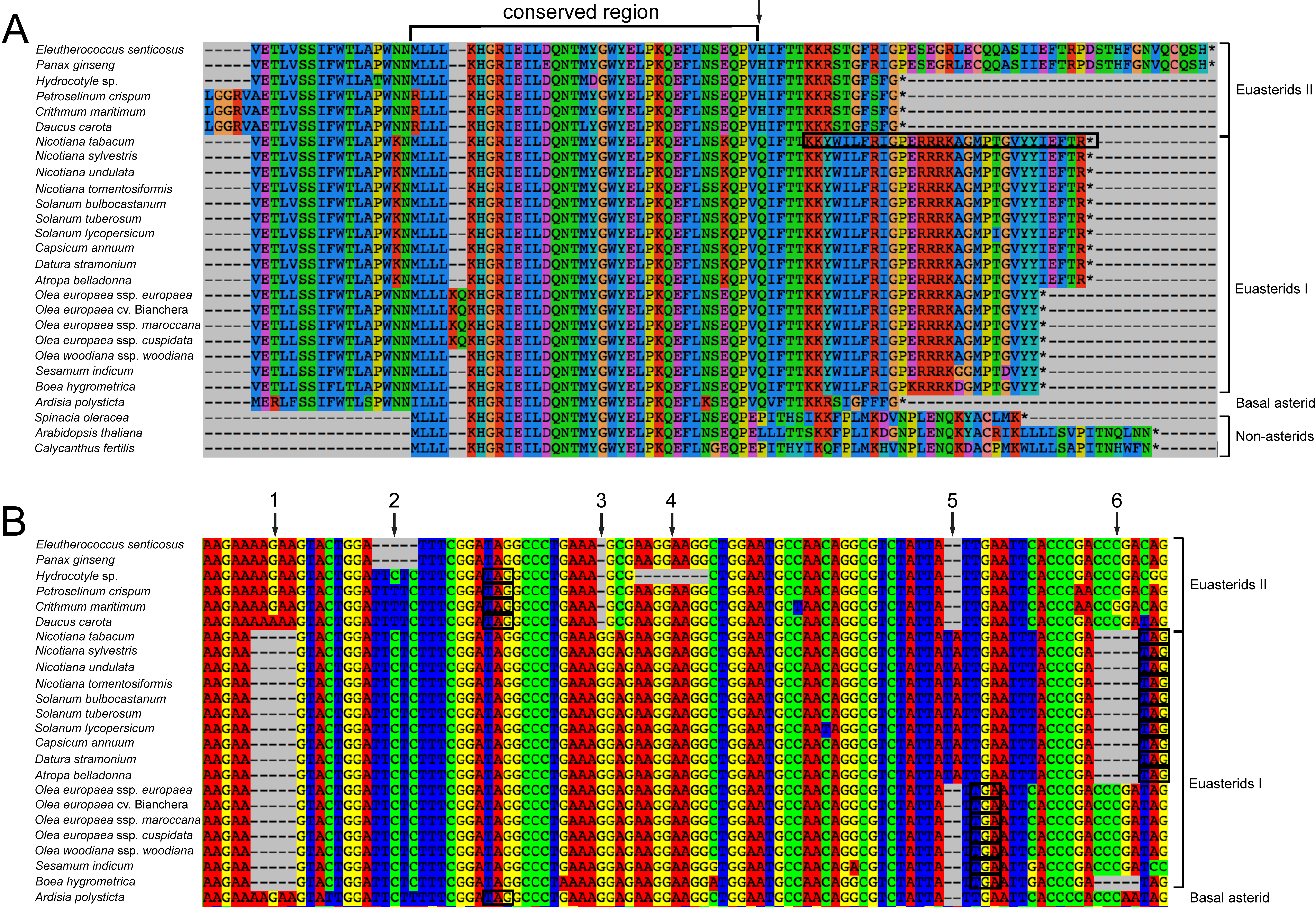

Supplement: Figure S2 — Alignment of ycf15 . A. Alignment of ycf15 amino acid sequences in Calycanthus, Arabidopsis, Spinacia, and asterids. The arrow indicates the divide between the 5′ and 3′ portions of ycf15 in Nicotiana tabacum, to which the homologous regions are separated by a 250–300 bp intervening sequence in non-asterid angiosperms. B. Alignment of asterid ycf15 nucleotide sequences corresponding the boxed region of Nicotiana tabacum in A. In-frame stop codons are boxed. Arrows indicate the six non-triplet indels. (TIF) [file pone.0062548.s002.tif]
