# Supplementary material for: Complete Plastid Genome Sequence of the Basal Asterid Ardisia polysticta Miq. and Comparative Analyses of Asterid Plastid Genomes
Source: PLoS One. 2013 Apr 30;8(4):e62548. doi: 10.1371/journal.pone.0062548 (PMC3640096; doi:10.1371/journal.pone.0062548)
Supplement: Table S1 — Accession numbers of complete plastome sequences of asterids and of those included in the phylogenetic tree in Figure 3 (bold). (DOCX) [file pone.0062548.s003.docx]

| Table S1. Accession numbers of complete plastome sequences of asterids and of those included in the phylogenetic tree in Figure 3 (bold). | | | | | | | |
| --- | --- | --- | --- | --- | --- | --- | --- |
| Higher taxa | Order | Family | Taxon | Size (bp) | GC (%) | No. of SSRs^a^ | Accession No. |
| Eurosids II | Brassicales | Brassicaceae | *Arabidopsis thaliana* | 154478 | 36.29 | 104 | NC_000932.1 |
| Caryophyllales | Caryophyllales | Amaranthaceae | *Spinacia oleracea* | 150725 | 36.82 | 65 | NC_002202.1 |
| Basal Asterid | Ericales | Myrsinaceae | *Ardisia polysticta* | 156506 | 37.07 | 57 | KC465962 |
| Euasterids I | Gentianales | Rubiaceae | *Coffea arabica* | 155189 | 37.43 | 43 | NC_008535.1 |
|  | Lamiales | Gesneriaceae | *Boea hygrometrica* | 153493 | 37.59 | 27 | NC_016468.1 |
|  |  | Oleaceae | *Jasminum nudiflorum* | 165121 | 37.98 | 68 | NC_008407.1 |
|  |  |  | *Olea europaea* cv. Bianchera | 155888 | 37.80 | 68 | NC_013707.2 |
|  |  |  | *Olea europaea* subsp. *cuspidata* | 155862 | 37.81 | 57 | NC_015604.1 |
|  |  |  | *Olea europaea* subsp. *europaea* cv. Manzanilla | 155875 | 37.81 | 65 | NC_015401.1 |
|  |  |  | *Olea europaea* subsp. *maroccana* | 155896 | 37.81 | 65 | NC_015623.1 |
|  |  |  | *Olea woodiana* subsp. *woodiana* | 155942 | 37.79 | 70 | NC_015608.1 |
|  |  | Orobanchaceae | *Epifagus virginiana* | 70028 | 36.00 | 52 | NC_001568.1 |
|  |  | Pedaliaceae | *Sesamum indicum* | 153324 | 38.20 | 35 | NC_016433.2 |
|  | Solanales | Convolvulaceae | *Cuscuta exaltata* | 125373 | 38.12 | 49 | NC_009963.1 |
|  |  |  | *Cuscuta gronovii* | 86744 | 37.72 | 29 | NC_009765.1 |
|  |  |  | *Cuscuta obtusiflora* | 85286 | 37.84 | 36 | NC_009949.1 |
|  |  |  | *Cuscuta reflexa* | 121521 | 38.22 | 48 | NC_009766.1 |
|  |  |  | *Ipomoea purpurea* | 162046 | 37.48 | 64 | NC_009808.1 |
|  |  | Solanaceae | *Atropa belladonna* | 156687 | 37.56 | 60 | NC_004561.1 |
|  |  |  | *Capsicum annuum* | 156781 | 37.73 | 46 | NC_018552.1 |
|  |  |  | *Datura stramonium* | 155871 | 37.88 | 49 | NC_018117.1 |
|  |  |  | *Nicotiana tabacum* | 155943 | 37.85 | 60 | NC_001879.2 |
|  |  |  | *Nicotiana tomentosiformis* | 155745 | 37.79 | 61 | NC_007602.1 |
|  |  |  | *Nicotiana sylvestris* | 155941 | 37.85 | 59 | NC_007500.1 |
|  |  |  | *Nicotiana undulata* | 155863 | 37.88 | 63 | NC_016068.1 |
|  |  |  | *Solanum bulbocastanum* | 155371 | 37.88 | 56 | NC_007943.1 |
|  |  |  | *Solanum lycopersicum* | 155461 | 37.86 | 56 | NC_007898.2 |
|  |  |  | *Solanum tuberosum* | 155296 | 37.88 | 53 | NC_008096.2 |
| Euasterids II | Apiales | Apiaceae | *Anthriscus cerefolium* | 154719 | 37.42 | 71 | NC_015113.1 |
|  |  |  | *Crithmum maritimum* | 158355 | 37.55 | 75 | NC_015804.1 |
|  |  |  | *Daucus carota* | 155911 | 37.66 | 67 | NC_008325.1 |
|  |  |  | *Oxypolis greenmanii* | 154737 | 37.32 | 61 | NC_015832.1 |
|  |  |  | *Petroselinum crispum* | 152890 | 37.78 | 60 | NC_015821.1 |
|  |  | Araliaceae | *Eleutherococcus senticosus* | 156768 | 37.95 | 42 | NC_016430.1 |
|  |  |  | *Hydrocotyle* sp. | 153207 | 37.59 | 46 | NC_015818.1 |
|  |  |  | *Panax ginseng* | 156318 | 38.11 | 37 | NC_006290.1 |
|  | Asterales | Asteraceae | *Ageratina adenophora* | 150698 | 37.46 | 33 | NC_015621.1 |
|  |  |  | *Guizotia abyssinica* | 151762 | 37.62 | 51 | NC_010601.1 |
|  |  |  | *Helianthus annuus* | 151104 | 37.62 | 52 | NC_007977.1 |
|  |  |  | *Jacobaea vulgaris* | 150689 | 37.32 | 58 | NC_015543.1 |
|  |  |  | *Lactuca sativa* | 152765 | 37.55 | 38 | NC_007578.1 |
|  |  |  | Parthenium argentatum | 152803 | 37.61 | 53 | NC_013553.1 |
|  |  | Campanulaceae | *Trachelium caeruleum* | 162321 | 38.33 | 57 | NC_010442.1 |
| ^a^Mono-, di-, tri-, tetra-, penta- and hexanucleotides with a length of at least 10 bp. | | | | | | | |
