# Supplementary material for: Complete Plastid Genome Sequence of the Basal Asterid Ardisia polysticta Miq. and Comparative Analyses of Asterid Plastid Genomes
Source: PLoS One. 2013 Apr 30;8(4):e62548. doi: 10.1371/journal.pone.0062548 (PMC3640096; doi:10.1371/journal.pone.0062548)
Supplement: Table S2 — Genes encoded in the Ardisia polysticta plastome. (DOCX) [file pone.0062548.s004.docx]

| **Table S2.** Genes encoded in the *Ardisia polysticta* plastome. | | |
| --- | --- | --- |
| Functional category | Group of genes | Gene name |
| Self-replication | rRNA genes | *rrn16*(×2), *rrn23*(×2), *rrn4.5*(×2), *rrn5*(×2), |
|  | tRNA genes | *trnA-UGC**(×2), *trnC-GCA*, *trnD-GUC*, *trnE-UUC*, *trnF-GAA*, *trnG-GCC*, *trnG-UCC**, *trnH-GUG*, *trnI-CAU*(×2), *trnI-GAU**(×2), *trnK-UUU**, *trnL-CAA*(×2), *trnL-UAA**, *trnL-UAG*, *trnfM-CAU*, *trnM-CAU*, *trnN-GUU*(×2), *trnP-UGG*, *trnQ-UUG*, *trnR-ACG*(×2), *trnR-UCU*, *trnS-GCU*, *trnS-GGA*, *trnS-UGA*, *trnT-GGU*, *trnT-UGU*, *trnV-GAC*(×2), *trnV-UAC**, *trnW-CCA*, *trnY-GUA* |
|  | Ribosomal small subunit | *rps2*, *rps3*, *rps4*, *rps7*(×2), *rps8*, *rps11*, *rps12*_*5’end*, *rps12*_*3’end**(×2), *rps14*, *rps15*, *rps16**, *rps18*, *rps19* |
|  | Ribosomal large subunit | *rpl2**(×2), *rpl14*, *rpl16**, *rpl20*, *rpl22*, *rpl23*(×2), *rpl32*, *rpl33*, *rpl36* |
|  | DNA-dependent RNA polymerase | *rpoA*, *rpoB*, *rpoC1**, *rpoC2* |
| Photosynthesis | Large subunit of rubisco | *rbcL* |
|  | Photosystem I | *psaA*, *psaB*, *psaC*, *psaI*, *psaJ*, *ycf3*** |
|  | Photosystem II | *psbA*, *psbB*, *psbC*, *psbD*, *psbE*, *psbF*, *psbH*, *psbI*, *psbJ*, *psbK*, *psbL*, *psbM*, *psbN*, *psbT*, *psbZ* |
|  | NADH dehydrogenase | *ndhA**, *ndhB**(×2), *ndhC*, *ndhD*, *ndhE*, *ndhF*, *ndhG*, *ndhH*, *ndhI*, *ndhJ*, *ndhK* |
|  | Cytochrome b/f complex | *petA*, *petB**, *petD**, *petG*, *petL*, *petN* |
|  | ATP synthase | *atpA*, *atpB*, *atpE*, *atpF**, *atpH*, *atpI* |
| Other | Maturase | *matK* |
|  | Subunit of acetyl-CoA carboxylase | *accD* |
|  | Envelope membrane protein | *cemA* |
|  | Protease | *clpP*** |
|  | Translational initiation factor | *infA* |
|  | c-type cytochrome synthesis | *ccsA* |
| Functions unknown | Conserved open reading frames (*ycf*) | *ycf1*, *ycf2*(×2), *ycf4*, *ycf15*(×2) |
| Note. The presence of one or two introns is indicated by one or two asterisks, respectively. Genes in the IR regions are followed by the (×2) symbol. | | |
|  |  |  |
