# Supplementary material for: Complete Plastid Genome Sequence of the Basal Asterid Ardisia polysticta Miq. and Comparative Analyses of Asterid Plastid Genomes
Source: PLoS One. 2013 Apr 30;8(4):e62548. doi: 10.1371/journal.pone.0062548 (PMC3640096; doi:10.1371/journal.pone.0062548)
Supplement: Table S3 — Sequences that correspond to the palindromic sequence in atpF-atpH in A. polysticta plastome (No. 5 in Table 4). (DOCX) [file pone.0062548.s005.docx]

| **Table S3.** Sequences that correspond to the palindromic sequence in *atpF*-*atpH* in *A*. *polysticta* plastome (No. 5 in Table 4). | |
| --- | --- |
| Taxa | Sequence |
| *Ardisia polysticta* | AAATATGAAAAAT**ACGT**ATTTTTCATATTT |
| *Jasminum nudiflorum* | AAATATGAAAAAT**AGAT**ATTTTTCATATTT |
| *Olea* spp. | AAATATGAAAAAT**ATGT**ATTTTTCATATTT |
| *Atropa belladonna* | AAATATGAAAAAT**GTTTTT**ATTTTTCATATTT |
| *Capsicum annuum* | AAATATGAAAAAT**ATATTT**ATTTTTCATATTT |
| *Datura stramonium* | AAATATGAAAAAT**GTATTT**ATTTTTCATATTT |
| *Nicotiana* spp. | AAATATGAAAAAT**GTATTT**ATTTTTCATATTT |
| *Solanum bulbocastanum* | AAATATGAAAAAT**AAATAT**ATTTTTCATATTT |
| *Solanum lycopersicum* | AAATATGAAAAAT**AT**ATTTTTCATATTT |
| *Solanum tuberosum* | AAATATGAAAAAT**AAATAT**ATTTTTCATATTT |
| *Eleutherococcus senticosus* | AAATAAGAAAAAT**TTGT**ATTTTTCATATTT |
| *Hydrocotyle* sp. | AAATATGAAAAAT**ATTT**CTTTTTCATATTT |
| *Panax ginseng* | AAATATGAAAAAT**ATTT**ATTTTTCATATTT |
| *Guizotia abyssinica* | AAATATGAAAAAT**TTTT**ATTTTTCATATTT |
| *Helianthus annuus* | AAATATGAAAAAT**TTGG**ATTTTTCATATTT |
| *Lactuca sativa* | AAATATGAAAAAT**CAAA**ATTTTTCATATTT |
| Parthenium argentatum | AAATATGAAAAAT**AAAA**ATTTTTCATATTT |
| Note. Loop positions are in bold. Stem positions different from those in *A*. *polysticta* are underlined. | |
