# Supplementary material for: Complete Plastid Genome Sequence of the Basal Asterid Ardisia polysticta Miq. and Comparative Analyses of Asterid Plastid Genomes
Source: PLoS One. 2013 Apr 30;8(4):e62548. doi: 10.1371/journal.pone.0062548 (PMC3640096; doi:10.1371/journal.pone.0062548)
Supplement: Table S4 — Types of plastome Inverted Repeat/Single Copy boundaries in asterids. (DOCX) [file pone.0062548.s006.docx]

| **Table S4.** Types of plastome Inverted Repeat/Single Copy boundaries in asterids. | |
| --- | --- |
| Types | Taxa |
| I-1: *rps19* on JLB, *ndhF* on JLB | *Ardisia*, *Boea*, *Hydrocotyle*, *Solanum* *tuberosum* |
| I-2: *rps19* on JLB, whole *ndhF* in SSC | *Coffea*, *Sesamum*, *Atropa*, *Capsicum*, *Datura*, *Nicotiana* *tomentosiformis*, *Solanum lycopersicon*, *Solanum* *bulbocastanum* *Eleutherococcus*, *Panax*, *Anthricus*, *Daucus*, *Oxypolis*, *Lactuca* |
| I-3: whole *rps19* in LSC, *ndhF* on JLB | *Olea* spp. |
| I-4: whole *rps19* in LSC, whole *ndhF* in SSC | *Nicotiana tabacum*, *Nicotiana* *sylvestris*, *Nicotiana* *undulata* |
| II: IR expansion to *rps15*-*ycf1* at JSA | *Jasminum* |
| III: IR contraction to *rpl23*-*trnI* at JLB, IR expansion to 5’ exon of *ndhA* at JSA | *Ipomoea* |
| IV: IR expansion to *rpl16*-*rps3* at JLB | *Crithmum* |
| V: IR contraction to 5’ exon of *rpl2* at JLB | *Petroselium* |
| VI: inversion of SSC, otherwise similar to Type II | *Helianthus*, *Guizotia*, *Parthenium*, *Ageratia*, *Jacobaea* |
| VII: multiple rearrangements within and among IR, LSC, SSC | *Trachelium* |
| Note. Parasitic asterids (*Epifagus virginiana* and *Cuscuta* spp.), which have undergone plastome reduction, are not included in this table. | |
